# Influence of the Reproductive and Non‐Reproductive Season on Hepatic Fat Accumulation in *Trachemys* sp.: Correlations of Computed Tomography, Histopathology, Biochemistry and Coelioscopy

**DOI:** 10.1111/ahe.70169

**Published:** 2026-08-26

**Authors:** Luna S. Rolim, Maria J. Mamprim, Sheila C. Rahal, Vivian F. Rech, Raphael A. B. Gonçalves, Jeana P. Silva, Fernanda B. C. Moura, Noeme S. Rocha

**Affiliations:** ^1^ Department of Veterinary Surgery and Animal Reproduction School of Veterinary Medicine and Animal Science, São Paulo State University (UNESP) Botucatu São Paulo Brazil; ^2^ Department of Veterinary Clinical Science, School of Veterinary Medicine and Animal Science São Paulo State University (UNESP) Botucatu São Paulo Brazil

**Keywords:** chelonians, diagnosis, energy metabolism, hepatic lipidosis

## Abstract

The hepatic energy storage of twenty‐four freshwater turtles was assessed using computed tomography, 3D Slicer software, serum biochemistry and histopathological analysis of liver biopsies to investigate the influence of seasonality and sex. In the evaluated animal group, seasonality did not influence hepatic fat and glycogen stores; however, significant sex‐related differences were observed. Female terrapins exhibited enlarged subcarapacial fat bodies, lower hepatic Hounsfield unit (HU) values and a greater degree of hepatocyte ballooning. Furthermore, females maintained higher serum concentrations of cholesterol, triglycerides, and calcium, particularly during the summer season. The values obtained in this study may be used as clinical reference values for adult male and female *Trachemys* spp.

## 1 Introduction

Several species of turtles, such as those belonging to the genus Trachemys, are commonly kept as pets. Because they are ectodermal animals, their maintenance requires special care regarding their environment and diet; however, handling errors are frequent and may compromise animal health (Divers et al. 2010). Liver diseases, such as lipidosis, are often associated with nutritional imbalance and inadequate environmental management. However, seasonal variations can also induce physiological changes in reptiles, which can make definitive diagnosis more difficult (Barboza et al. 2023; Beaufrere et al. 2024).

This study aimed to investigate the influence of the reproductive and non‐reproductive seasons on hepatic fat accumulation in freshwater turtles. In addition, the study sought to evaluate the sensitivity of computed tomography (CT) for identifying this variation and to assess how this imaging technique can be used as an effective diagnostic tool. Furthermore, correlations among CT findings, serum biochemistry and hepatic histopathology were evaluated to determine whether low or negative hepatic HU values were associated with increased liver enzyme activities, greater hepatic fat accumulation, and hepatocellular injury.

## 2 Materials and Methods

A cross‐sectional study was conducted on twenty‐four adult captive Trachemys sp. turtles, 10 males and 14 females, from São Paulo, Brazil.

Evaluations were performed during two distinct periods: from June to August, corresponding to the dry winter season and non‐reproductive period, and from December to February, corresponding to the wet summer season and reproductive period. The evaluation periods were established based on a literature review regarding seasonal influences on reproduction and activity of Brazilian chelonian species (Silva et al. 1984; Thomas et al. 1999; Fagundes et al. 2010; Gradela et al. 2020).

The study was approved by the School of Veterinary Medicine and Animal Science Institutional Ethics Committee for Animal Use (CEUA 0350/2023).

### 2.1 Animal Selection

Adult animals of both sexes, as determined by typical morphological characteristics, and that were free of any clinical signs of illness were included in this evaluation.

The animals were monitored daily between 2023 and 2025, and body mass was recorded on a quarterly basis to obtain data on weight gain and carapace growth. Animals showing evidence of lesions or debilitation were excluded from the study. All turtles were fed a commercial freshwater turtle pellet diet, and no dietary modifications were made during this period.

### 2.2 Advanced Imaging Examination

Computed tomography (CT) was performed with the animals positioned in ventral recumbency. A drape was wrapped around the tarsus region to keep the pelvic limbs together and stabilize the carapace on the CT table. The procedure was performed under mild sedation with dexmedetomidine (0.02 mg/kg).

Images were acquired using a Shimadzu SCT‐7800 CT scanner (Shimadzu, Kyoto, Japan) calibrated at 120 kVp and 130 mA. Cross‐sectional images were reconstructed using multiplanar reconstruction (MPR). Regions of interest (ROIs) were selected for densitometric evaluation, avoiding vascular structures (King et al. 2019). Twelve ROIs were selected per animal, with two in each liver lobe (right and left, ventral and dorsal) across three image slices, each measuring 20 mm^2^. Image assessment was conducted using the PACS system (Synapse, Fuji Medical System, Tokyo, Japan).

Mean attenuation values (HU) of hepatic parenchyma were calculated for each animal, and these values were then compared with serum and histopathological results.

The percentage of body fat was calculated using 3D Slicer software after three‐dimensional reconstruction, applying a threshold between −10 and −110 HU (Walden et al. 2022).

### 2.3 Haematological and Serum Biochemistry Evaluation

Blood samples were collected from the jugular vein, stored in heparinized tubes, and processed within 1 h. Animals were fasted for 48 h before blood sampling.

Haematological analyses included packed cell volume (PCV), total erythrocyte and leukocyte counts that were performed manually in a Neubauer chamber and Natt‐Herrick diluent. Blood smears were stained using panoptic stain for differential leukocyte counts, including heterophils, basophils, lymphocytes, eosinophils and thrombocytes (Campbell 2012; Sykes and Klaphake 2015).

Serum biochemistry was performed to determine aspartate aminotransferase (AST), total calcium (Ca2+), phosphorus (P), total protein (TP), albumin (ALB), globulins (GLOB), cholesterol (CHOL) and triglycerides (TG) using an enzymatic colorimetric method (EBRAM).

### 2.4 Hepatic Biopsy via Laparoscopy

The procedure was performed in a temperature‐controlled environment (27°C), following a 48‐h fasting period. The sedation was achieved using ketamine (10 mg/kg), dexmedetomidine (0.1 mg/kg) and morphine (1 mg/kg) administered intramuscularly. A jugular venous catheter was placed, and general anaesthesia was induced using propofol (5 mg/kg IV) and maintained with isoflurane (2% in oxygen). Analgesia was supplemented with intrathecal lidocaine (4 mg/kg) and tramadol (5 mg/kg IM every 12 h for 2 days postoperatively).

Laparoscopy was performed from femoral access to collect liver biopsies using a 2 mm endoscopic biopsy forceps (Schildger et al. 1999). Samples were obtained from both liver lobes, fixed in 10% buffered formalin for histopathological evaluation.

### 2.5 Histopathology of Hepatic Tissue

Liver biopsy samples were routinely processed for paraffin embedding and stained with haematoxylin and eosin (H&E) for histological evaluation, including assessment of hepatocellular vacuolization. To assess hepatic fibrosis, Masson's trichrome stain was utilized, while Periodic Acid‐Schiff (PAS) stain was employed for glycogen deposition analysis. All slides were independently reviewed in a double‐blind manner by two veterinary pathologists.

### 2.6 Statistical Analysis

Statistical analyses were performed using GraphPad Prism software version 8.0. Data normality was verified using the Shapiro–Wilk test. For paired parametric data, Student's *t*‐test was applied, whereas the Wilcoxon signed‐rank test was used for paired nonparametric data.

Sex‐related comparisons were performed using Student's *t*‐test for independent samples, with Welch's correction when variances were heterogeneous. The Mann–Whitney test was used for nonparametric data.

Correlations between variables were evaluated using Pearson's correlation test for linearly distributed data or Spearman's correlation test for nonparametric data.

All statistical tests were two‐tailed, and results were considered statistically significant when *p* < 0.05.

## 3 Results

Fifteen turtles (eight females and seven males of the genus Trachemys sp.) were evaluated during both seasonal periods (summer and winter). Females had a significantly greater body mass than males (*p* = 0.0067), with a mean body mass of 1.68 kg (range, 1.10–2.75 kg) compared with 0.85 kg (range, 0.70–1.10 kg) in males.

Haematological comparison between seasons demonstrated significantly higher mean corpuscular volume (MCV) (*p* = 0.0147) and total plasma protein (TPP) (*p* = 0.0078) values during winter. Male turtles also exhibited significantly higher haemoglobin (*p* = 0.0156) and haematocrit (*p* = 0.0218) values during winter, whereas no significant differences were observed for other haematological parameters.

Biochemical analyses demonstrated significant sex‐related differences. Females presented significantly higher serum triglyceride (*p* = 0.0121), cholesterol (*p* = 0.0059) and calcium (*p* = 0.0126), although no significant seasonal differences were identified.

**FIGURE 1:**
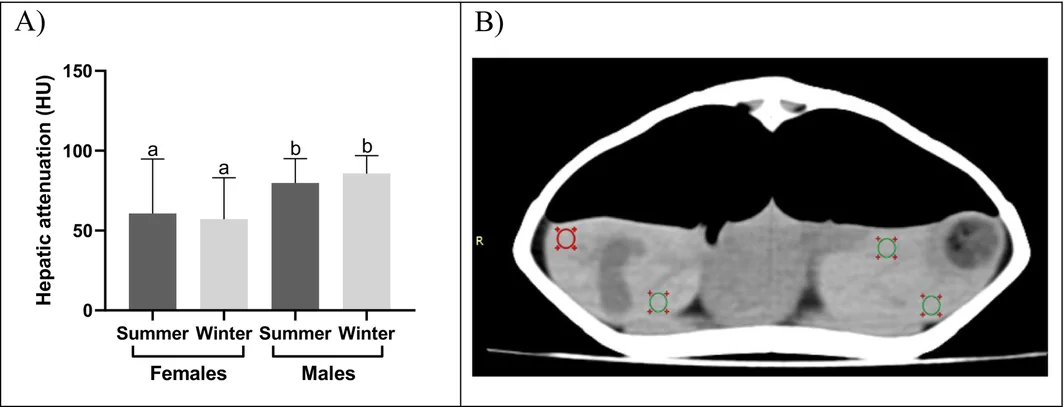
Measurement of hepatic attenuation values (HU) in freshwater turtles (*Trachemys* sp.). (A) Comparative analysis of hepatic attenuation in males and females during the reproductive (summer) and non‐reproductive (winter) seasons. (B) Transverse CT image of the coelomic cavity demonstrating regions of interest (ROIs) positioned within the right and left hepatic lobes.

**FIGURE 2:**
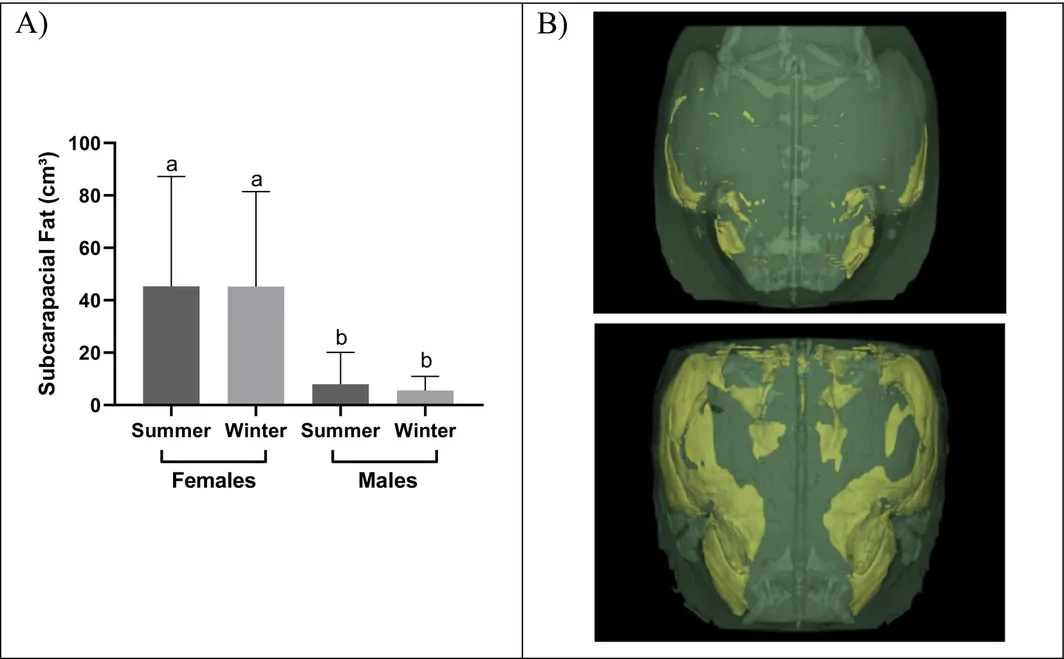
Comparative analysis of fat accumulation in male and female freshwater turtles (*Trachemys* sp.). (A) Hepatic HU values during the reproductive season (summer) and non‐reproductive season (winter). (B) Dorsal view of reconstructed computed tomography images visualized in 3D Slicer, with the carapace shown in green and the fat distribution highlighted in yellow; the male turtle is displayed in the upper panel and the female in the lower panel.

Tomographic evaluation revealed no significant seasonal differences in hepatic attenuation (HU). However, females exhibited significantly lower hepatic attenuation values than males (*p* = 0.0182), as shown in Figure 1. Females also showed significantly greater subcarapacial fat accumulation (45.33 cm^3^, corresponding to 4% of total carapace volume) than males (7.95 cm^3^, corresponding to 1.25%) (*p* = 0.0022). During winter, males demonstrated a slight, non‐statistically significant increase in subcarapacial fat (Figure 2).

Tomographic correlations demonstrated a strong negative correlation between the percentage of subcarapacial fat and hepatic HU (Rho = −0.631; *p* < 0.001).

Macroscopic hepatic evaluation by celioscopy demonstrated seasonal differences in liver colour and size. During summer, 20% of the livers exhibited increased volume and yellowish discoloration, whereas during winter, 80% of the livers appeared macroscopically normal.

**FIGURE 3:**
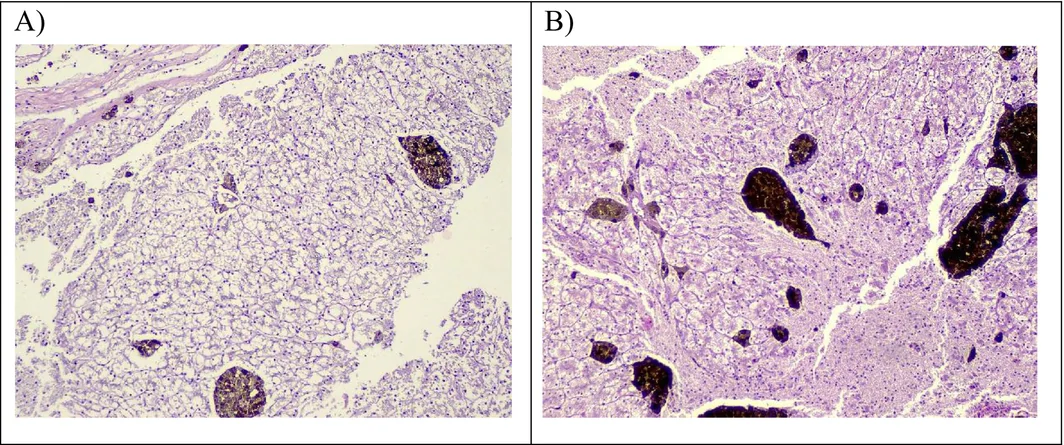
Photomicrographs of the hepatic parenchyma of a female Trachemys sp., stained with haematoxylin and eosin (H&E), magnification 100X. An increase in hepatocyte ballooning is noted in summer (A) compared to winter (B).

Histological evaluation revealed a significant increase in hepatic ballooning in summer, particularly in females (*p* = 0.0020), although vacuolization was generally mild or absent (Figure 3). Glycogen deposition and fibrosis were identified in both seasons, without significant differences between sexes and seasons.

Correlation analyses demonstrated associations among biochemical, tomographic and histopathological findings. A strong positive correlation was observed between subcarapacial fat percentage and triglyceride concentrations (Rho = 0.665; *p* < 0.001), as well as cholesterol concentrations (Rho = 0.649; *p* < 0.001).

Histopathological findings showed moderate correlations with biochemical and tomographic variables, particularly between hepatocyte ballooning and subcarapacial fat percentage (Rho = −0.511; *p* < 0.001), suggesting an association between lipid accumulation and hepatic morphological alterations.

Aspartate aminotransferase (AST) activity demonstrated a weak positive correlation with hepatic fibrosis (Rho = 0.364; *p* = 0.011) and was elevated in all animals with moderate to severe fibrosis.

## 4 Discussion

The present findings demonstrate significant physiological differences between male and female Trachemys sp., particularly regarding biochemical parameters and energy storage, which are likely associated with the female reproductive cycle (Duggan et al. 2001; Blanvillain et al. 2011).

The elevated triglycerides, cholesterol and calcium concentrations observed in females are consistent with previous studies (Deem et al. 2009; Innis and Knotek 2020; DiGirolamo 2022), which associate these changes with vitellogenesis and oestrogen‐mediated mobilization of lipids and minerals required for egg production.

The absence of significant variations in hepatic HU values between seasons suggests that seasonality did not directly impact liver density. However, the lower HU values observed in females may reflect greater hepatic lipid accumulation associated with vitellogenesis. The negative correlation between hepatic HU values and subcarapacial fat supports this hypothesis, indicating that animals with greater fat accumulation exhibited lower hepatic density on CT examination.

Histopathological findings support this interpretation, as increased hepatocyte ballooning was observed during summer. Nevertheless, the absence of marked cytoplasmic vacuolization may also indicate hydropic degeneration rather than lipid accumulation alone. Additional lipid‐specific histochemical staining techniques would be necessary to differentiate these processes.

The presence of fibrosis and glycogen deposition in both periods, without significant variations, suggests that these findings may represent common features in adult turtles rather than season‐dependent alterations in these species.

The correlations observed among biochemical, tomographic and histopathological findings indicate that CT, particularly the assessment of hepatic attenuation and subcarapacial fat volume, may represent a useful noninvasive tool for evaluating metabolic and hepatic status in freshwater turtles.

The association between reduced hepatic HU and increased serum lipid concentrations, fat accumulation, hepatocyte ballooning and vacuolization suggests an adaptive process linked to reproduction. However, these findings may also indicate a predisposition to subclinical hepatic steatosis in captive freshwater turtles.

## 5 Conclusion

The results of this study indicate that the liver physiology of turtles of the genus Trachemys sp. is significantly influenced by sex and, to a lesser extent, by seasonality. Females exhibited greater subcarapacial fat accumulation and hepatic changes consistent with the reproductive period, suggesting that reproductive activity directly affects lipid metabolism and liver morphology.

Computed tomography proved effective in identifying these alterations, particularly through the assessment of hepatic attenuation and body fat volume, which correlated with biochemical and histopathological findings. These findings highlight the potential of CT as a complementary diagnostic tool for evaluating metabolic alterations in turtles and for the early detection of hepatic steatosis, although CT alone remains insufficient for definitive diagnosis.

The integration of imaging, biochemical, and histopathological analyses is essential for a more accurate and preventive approach in reptile medicine.

## Author Contributions

Conception and design: Luna S. Rolim and Maria J. Mamprim. Acquisition of data: Luna S. Rolim, Vivian F. Rech and Jeana P. Silva. Analysis and interpretation of data: Luna S. Rolim, Fernanda B.C. Moura, Noeme S. Rocha and Maria J. Mamprim. Drafting the article: Luna S. Rolim, Maria J. Mamprim and Raphael A.B. Gonçalves. Review and editing – English language: Sheila C. Rahal. Final approval of the completed article: Luna S. Rolim, Maria J. Mamprim, Sheila C. Rahal, Raphael A.B. Gonçalves and Jeana P. Silva.

## Funding

This work was supported by Coordenação de Aperfeiçoamento de Pessoal de Nível Superior. Financiadora de Estudos e Projetos, 01.12.0530.00.

## Conflicts of Interest

The authors declare no conflicts of interest.

## Data Availability

The data that support the findings of this study are available from the corresponding author upon reasonable request.
